# Effects of treatment with SGLT-2 inhibitors on arginine-related cardiovascular and renal biomarkers

**DOI:** 10.1186/s12933-021-01436-x

**Published:** 2022-01-06

**Authors:** Arne Gessner, Anna Gemeinhardt, Agnes Bosch, Dennis Kannenkeril, Christian Staerk, Andreas Mayr, Martin F. Fromm, Roland E. Schmieder, Renke Maas

**Affiliations:** 1 Institute of Experimental and Clinical Pharmacology and Toxicology, Friedrich-Alexander-Universität Erlangen-Nürnberg, Fahrstr. 17, 91054 Erlangen, Germany; 2 Department of Nephrology and Hypertension, University Hospital Erlangen, Erlangen, Germany; 3 Institute of Medical Biometry, Informatics and Epidemiology, University Hospital Bonn, Bonn, Germany

**Keywords:** SGLT-2 inhibitor, Empagliflozin, Dapagliflozin, Type 2 diabetes mellitus, Homoarginine, ADMA, SDMA, Cardiovascular disease

## Abstract

**Background:**

In patients with type 2 diabetes (T2D) sodium-glucose cotransporter 2 (SGLT-2) inhibitors improve glycaemic control as well as cardiovascular and renal outcomes. Their effects on l-arginine (Arg) related risk markers asymmetric and symmetric dimethylarginine (ADMA and SDMA) and the protective biomarker L-homoarginine (hArg) linking T2D to cardiovascular and renal disease have not yet been reported.

**Methods:**

Plasma and 24-h urine samples taken before and after 6 weeks of treatment were available from two prospective, randomized, double-blind, placebo-controlled, cross-over trials with empagliflozin (71 patients analyzed, NCT02471963) and dapagliflozin (59 patients analyzed, NCT02383238). In these samples, concentrations of hArg, Arg, ADMA, SDMA, and creatinine were determined by liquid-chromatography coupled to tandem mass-spectrometry. Additionally, intraindividual changes of the biomarkers in plasma were correlated with intraindividual changes of clinical parameters.

**Results:**

Treatment with empagliflozin and dapagliflozin was associated with a reduction of plasma hArg by 17.5% and 13.7% (both p < 0.001), respectively, and increase in plasma SDMA concentration of 6.7% and 3.6%, respectively (p < 0.001 and p < 0.05), while plasma Arg and ADMA concentrations were not significantly altered. 24-h urinary excretion of ADMA was reduced by 15.2% after treatment with empagliflozin (p < 0.001) but not after dapagliflozin treatment, while excretion of the other markers was not significantly altered. Renal clearance of SDMA was reduced by 9.1% and 3.9% for both drugs (both p < 0.05). A reduction in ADMA clearance was observable after empagliflozin treatment only (− 15.5%, p < 0.001), but not after dapagliflozin. Renal clearance of hArg and Arg was not significantly altered. Treatment effects on l-arginine related biomarkers were not constantly correlated with effects on glycated hemoglobin, fasting plasma glucose, body mass index, and systolic blood pressure.

**Conclusions:**

Treatment with SGLT-2 inhibitors has divergent effects on Arg-related biomarkers and could affect risk estimates associated with these markers. The observed effects are unlikely to explain the known cardiovascular and renal benefits of treatment with empagliflozin or dapagliflozin but still may indicate new therapeutic approaches in patients treated with SGLT-2 inhibitors.

*Trial registration* http://www.clinicaltrials.gov: NCT02471963 (registered 15th June 2015, retrospectively registered) and NCT02383238.

**Supplementary Information:**

The online version contains supplementary material available at 10.1186/s12933-021-01436-x.

## Background

Type 2 diabetes mellitus (T2D) is a risk factor for cardiovascular disease (CVD) and chronic kidney disease (CKD), both contributing to the increased mortality risk in T2D, especially when diabetes is poorly controlled [1, 2]. Consequently, treatment of T2D frequently aims not only to control hyperglycaemia, but also to reduce cardiovascular and renal sequelae. Inhibition of the sodium-glucose cotransporter 2 (SGLT-2), e.g. via drugs like empagliflozin and dapagliflozin, has been shown to be a very effective treatment option in T2D. SGLT-2 inhibitors not only improved glycaemic control by increased renal excretion of glucose, but also protected from cardiovascular events and decline of renal function [3–9].

It remains to be elucidated whether the beneficial effects of SGLT-2 inhibitors can simply be attributed to their general glucose lowering effect, or at least in part also to their specific diuretic mode of action, which includes possible effects on other renally excreted risk markers and risk factors. Of special interest in this respect are risk markers commonly affected in T2D as well as in CVD and CKD. Among others, all three conditions are associated with endothelial dysfunction and altered l-arginine-NO signalling, as well as altered plasma concentrations of the l-arginine (Arg) related biomarkers L-homoarginine (hArg), asymmetric and symmetric dimethylarginine (ADMA and SDMA). Arg can be utilized by nitric oxide synthase to generate nitric oxide and thereby impact endothelial function [10], this makes Arg a potential protective substance in terms of CVD or CKD. For hArg a predictive value as a protective marker is well established, as several studies found it to be independently associated with positive outcomes on cardiovascular and renal function [11–15]. ADMA and SDMA on the other hand, have been studied as risk markers [10, 16–20]. Interactions with nitric oxide synthase are being discussed as putative mechanisms for the positive (Arg, hArg) or negative (ADMA, SDMA) association with clinical outcomes, however, the exact mechanisms are still elusive. As these markers are structurally and functionally interrelated, but also differ in their metabolism and/or renal handling, we hypothesised that an investigation of the effects of SGLT-2 inhibition with empagliflozin or dapagliflozin on the plasma concentration and renal excretion of Arg-related biomarkers may offer new mechanistic insights beyond the blood glucose lowering effect of the drugs.

In the present study we evaluated the effects of SGLT-2 inhibition with empagliflozin and dapagliflozin on plasma concentrations and renal excretion of Arg and its derivatives hArg, ADMA and SDMA in plasma and 24-h-urine samples from two recently completed randomized double-blind, placebo-controlled, cross-over trials [21, 22].

## Methods

### Study design

This study combines data and samples from two prospective, randomized, double-blind, placebo-controlled, cross-over trials of empagliflozin and dapagliflozin in patients with T2D (NCT02471963 and NCT02383238). The detailed methods and patient characteristics of the two trials have been previously published [21–23]. The study design is shown in Additional file 1: Figure S1. In brief, in both studies patients were randomized after a run-in/washout period to receive either once-daily oral empagliflozin 25 mg/dapagliflozin 10 mg or placebo for six weeks followed by a week of washout and then switched to either placebo or verum for another six weeks. Blood and 24-h urine samples were collected at baseline, after 6 weeks with placebo, and after 6 weeks with empagliflozin or dapagliflozin. At the indicated time points plasma samples were taken and urine was collected over 24 h. All samples were stored at − 80 °C after collection was completed and thawed immediately before analysis. Study protocols were approved by German drug authorities, as well as by the local ethics committee and written informed consent was obtained.

### Chemicals and materials

All mass-labeled and unlabeled reference standards were in the L-form. Creatinine-HCl, Arg, and hArg-HCl, were purchased from Sigma-Aldrich (Darmstadt, Germany). SDMA-HCl and ADMA-HCl were purchased from Enzo Life Sciences (Lörrach, Germany). [^2^H_3_]-creatinine was purchased from CDN-isotopes (Pointe-Claire, QC, Canada). [^2^H_7_]-Arg, [^13^C_7_^15^N_4_]-hArg-HCl, [^2^H_6_]-SDMA-HCl, and [^2^H_7_]-ADMA were purchased from Cambridge Isotope Laboratories (Tewksbury, MA, USA). All standards had a purity of > 98%. Acetonitrile, water and formic acid (all LC/MS-grade) were purchased from VWR chemicals (Darmstadt, Germany). Ammonium formate (LC/MS-grade) was purchased from Sigma-Aldrich (Darmstadt, Germany).

### Analysis of plasma and urine samples

From 71 patients of the empagliflozin study and from 59 patients of the dapagliflozin study plasma samples were available for the time points studied, and from 71 patients of the empagliflozin study and from 59 patients of the dapagliflozin study urine samples were available for the time points studied.

All samples were analyzed by a validated liquid-chromatography coupled to tandem mass-spectrometry assay adapted from a previously published method [24]. In brief, 20 µL of sample were mixed with 100 µL of acetonitrile containing deuterated internal standards of the analytes of interest. After centrifugation, 100 µL of supernatant were diluted with 300 µL of chromatographic eluent. Analysis was carried out on an HPLC system (Agilent 1100; Agilent Technologies, Waldbronn, Germany) coupled to a Triple-Quad-MS (API 4000; Applied Biosystems, Darmstadt, Germany) featured with an electrospray source operated in positive mode. Chromatography was carried out isocratically at a flow rate of 0.25 mL/min. The eluent was a mixture of 4 g/L ammonium formate in acetonitrile–water (75:25), which was adjusted to pH 4 with formic acid. The column was an EC 150/2 Nucleoshell HILIC column (2.7 μm, 150 × 2 mm) equipped with a guard column (4 × 2 mm) (both from Macherey–Nagel, Düren, Germany). The column temperature was set to 30 °C and the run time to 11 min. A dynamic MRM method was applied for mass spectrometric detection of the analytes.

### Statistics and calculation of renal clearance

Data are shown as absolute values or percentages, both in means ± standard deviation (SD), unless otherwise indicated. IBM SPSS Statistics (Version 25) and R (Version 4.0.5) were used for statistical analyses. A two-sided p-value of < 0.05 indicated statistical significance. Renal clearance (Cl_R_) was calculated by using the determined concentrations in plasma and urine, along with the volume of the urine collected over 24 h, as follows: (c_urine_*V_urine_)/(c_plasma_*1440 min).

Two-sided paired t-tests or two-sided Wilcoxon signed-rank tests were used to investigate differences in biomarkers between placebo and verum, between placebo and baseline, as well as between verum and baseline (considering absolute and percentage changes from baseline), respectively. Additionally, adjusted p-values for multiple testing were computed based on the procedure of Benjamini and Hochberg controlling the false discovery rate [25].

In sensitivity analyses linear mixed effects models were used to investigate differences in post-baseline concentrations of biomarkers (after verum and after placebo), adjusting for concentrations at baseline and the ordering in the cross-over design. Subject-specific random intercepts were included to account for repeated measurements, while age and sex were included as additional fixed effects. For right-skewed biomarkers (urine concentration and renal clearance), a log-transformation was applied for statistical inference. Effect estimates of linear mixed models refer to adjusted differences in (log) concentrations between verum and placebo, which are reported with corresponding two-sided 95% confidence intervals and p-values. In a further exploratory analysis, Pearson correlations were calculated between, on the one hand, the absolute changes in the plasma concentration from baseline to after verum treatment for hArg, Arg, ADMA, and SDMA, and on the other hand, changes in glycated haemoglobin (HbA1c), fasting plasma glucose (FPG), body mass index (BMI), and systolic blood pressure (SBP), respectively.

## Results

### Patients

Detailed patient characteristics of the two underlying clinical trials have been previously published [21–23]. Key characteristics are briefly presented again for reference. In the empagliflozin trial the mean age was 62 years and 59% of the patients were male, in the dapagliflozin trial the mean age was 60 years and 61% of the patients were male. Effects of both SGLT-2 inhibitors on HbA1c, FPG, BMI, and SBP are shown in Additional file 1: Table S2.

### Plasma concentrations of arginine-related biomarkers

Results for biomarkers in plasma are summarized in Table 1 and Additional file 1: Table S4.

**Table 1:** Plasma concentrations of biomarkers after empagliflozin/dapagliflozin treatment

	At baseline	After placebo		After verum			Reference values in healthy populations (2.5th–97.5th centile) [32]
			p-value [adj. p-value] vs. baseline (n)		p-value [adj. p-value] vs. baseline (n)	p-value [adj. p-value] vs. placebo (n)	
*Empagliflozin*							
Homoarginine	1.667 ± 0.589	1.631 ± 0.593	0.484 [0.660] (71)	1.376 ± 0.481	< 0.001 [0.027] (71)	< 0.001 [< 0.001] (71)	1.4–2.6
Arginine	70.44 ± 16.82	71.63 ± 14.25	0.539 [0.705] (71)	68.27 ± 19.32	0.292 [0.453] (71)	0.122 [0.261] (71)	41–114
ADMA	0.434 ± 0.062	0.429 ± 0.064	0.476 [0.660] (71)	0.436 ± 0.056	0.768 [0.863] (71)	0.338 [0.507] (71)	0.41–0.96
SDMA	0.371 ± 0.072	0.355 ± 0.062	0.008 [0.028] (71)	0.396 ± 0.076	< 0.001 [< 0.001] (71)	< 0.001 [0.002] (71)	0.27–0.67
Creatinine	65.35 ± 13.04	65.57 ± 12.69	0.808 [0.866] (71)	67.11 ± 14.18	0.055 [0.146] (71)	0.050 [0.141] (71)	n.a
*Dapagliflozin*							
Homoarginine	1.593 ± 0.555	1.581 ± 0.589	0.899 [0.963] (57)	1.375 ± 0.465	< 0.001 [0.007] (59)	< 0.001 [0.007] (57)	1.4–2.6
Arginine	72.60 ± 20.23	70.79 ± 19.16	0.635 [0.774] (57)	71.70 ± 19.17	0.739 [0.831] (59)	0.980 [0.984] (57)	41–114
ADMA	0.448 ± 0.057	0.423 ± 0.072	0.003 [0.034] (57)	0.429 ± 0.066	0.021 [0.090] (59)	0.365 [0.604] (57)	0.41–0.96
SDMA	0.364 ± 0.068	0.351 ± 0.073	0.061 [0.153] (57)	0.377 ± 0.076	0.026 [0.090] (59)	< 0.001 [0.007] (57)	0.27–0.67
Creatinine	66.13 ± 13.94	65.35 ± 15.47	0.706 [0.831] (57)	68.25 ± 14.85	0.040 [0.120] (59)	0.053 [0.140] (57)	n.a

Data are given in µM as mean ± SD, n.a.: not applicable; p-values calculated by two-sided paired t-tests, adj. p-values computed based on the procedure of Benjamini and Hochberg; p-values refer to the absolute changes in biomarker concentration

**Fig. 1:**
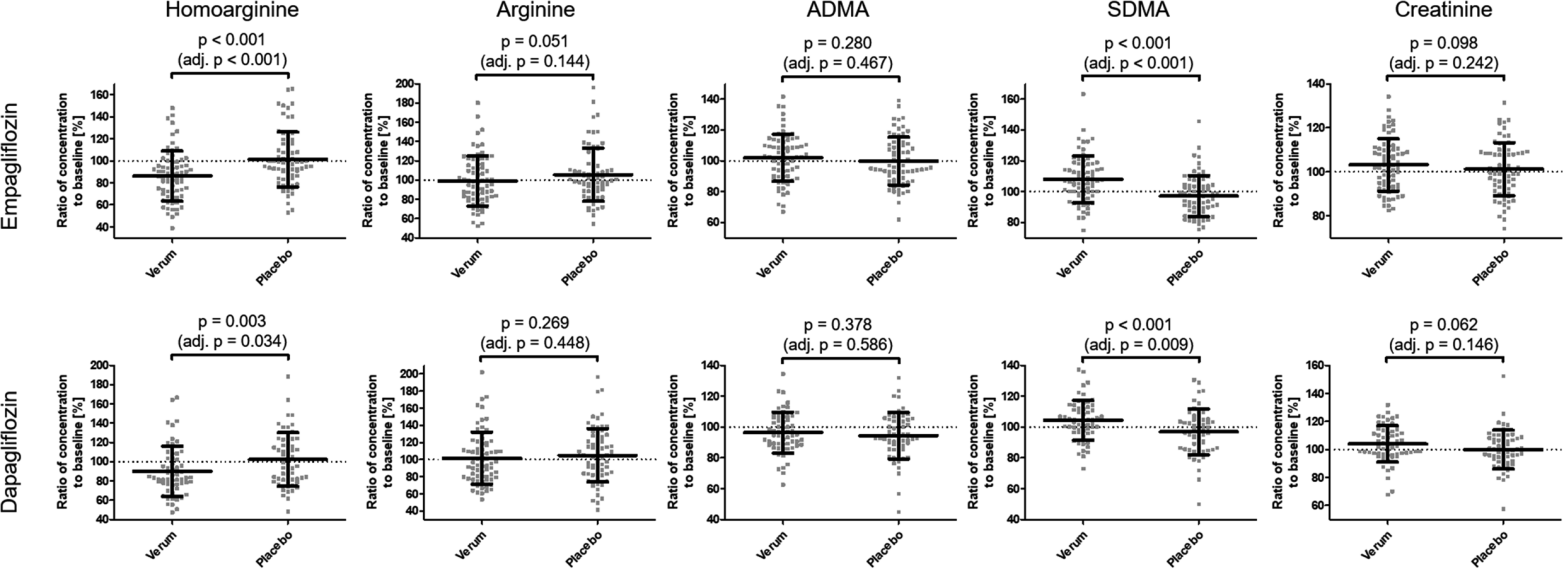
Intraindividual percentage ratios after treatment with empagliflozin or dapagliflozin in comparison to baseline values (Verum) and after placebo treatment in comparison to baseline values (Placebo) for plasma concentration of hArg, Arg, ADMA, SDMA, and creatinine. P-values calculated by two-sided paired t-tests; p-values refer to the percentage changes of biomarkers; adj. p-values computed based on the procedure of Benjamini and Hochberg

In comparison to baseline values, treatment with empagliflozin resulted in hArg plasma concentration to be decreased by a mean of 0.291 µM after empagliflozin (p < 0.001 [adj. p = 0.027]), and 0.036 µM after placebo (p = 0.484 [adj. p = 0.660]). Mean intraindividual percentage changes in comparison to baseline were − 13.9% after empagliflozin treatment and + 1.2% after placebo, with a p-value of p < 0.001 [adj. p < 0.001] between empagliflozin and placebo (Fig. 1). Plasma concentration of hArg after treatment with dapagliflozin was reduced by a mean of 0.218 µM after dapagliflozin (p < 0.001 [adj. p = 0.007]) and 0.012 µM after placebo (p = 0.899 [adj. p = 0.963]) compared to values at baseline. Mean intraindividual changes compared to baseline were − 10.0% after dapagliflozin and + 2.2% after placebo, with a p-value of 0.003 [adj. p = 0.034] between them (Fig. 1). Linear mixed effects models showed similar treatment effects for both drugs (Additional file 1: Table S4).

We detected no significant changes in plasma concentrations of Arg and ADMA.

Plasma samples in the empagliflozin study had a mean increase of SDMA concentration from baseline of 0.025 µM after verum (p < 0.001 [adj. p < 0.001]). After placebo treatment concentrations of SDMA in plasma were 0.016 µM lower than at baseline (p = 0.008 [adj. p = 0.028]). Mean intraindividual changes compared to baseline were + 8.0% after empagliflozin and -2.8% after placebo, with a p-value of p < 0.001 [adj. p < 0.001] between them (Fig. 1). Likewise, in the dapagliflozin study SDMA plasma concentrations increased by 0.013 µM after dapagliflozin (p = 0.026 [adj. p = 0.090]) and were reduced by 0.013 µM after placebo (p = 0.061 [adj. p = 0.153]) compared to baseline. Intraindividual changes were + 4.3% after dapagliflozin and − 3.2% after placebo (p < 0.001 [adj. p = 0.009]) (Fig. 1). Linear mixed effects models showed a similar effect of verum treatment in both studies (Additional file 1: Table S4).

Creatinine plasma concentration was increased by a mean of 1.76 µM after verum (p = 0.055 [adj. p = 0.146]) and 0.22 µM after placebo (p = 0.808 [adj. p = 0.866]) in the empagliflozin trial. The intraindividual percentage increase from baseline values was 3.1% after empagliflozin and 1.2% after placebo (p = 0.098 [adj. p = 0.242]). In the dapagliflozin trial the mean increase was 2.12 µM after dapagliflozin (p = 0.040 [adj. p = 0.120]) and the mean reduction after placebo was 0.78 µM (p = 0.706 [adj. p = 0.831]). The intraindividual changes were + 3.9% for dapagliflozin and − 0.1% for placebo (p = 0.062 [adj. p = 0.146] between them) as shown in Fig. 1. Linear mixed effects models showed a comparable increase of creatinine plasma concentrations after verum treatment with respect to placebo treatment for both empagliflozin and dapagliflozin (Additional file 1: Table S4).

### Renal excretion of biomarkers

Table 2, as well as Additional file 1: Table S5 and Fig. S2 summarize characteristics of renal excretion for the Arg-related biomarkers after treatment with empagliflozin and dapagliflozin.

**Table 2:** Amount excreted in urine over 24 h of biomarkers after empagliflozin/dapagliflozin treatment

	At baseline	After placebo		After verum		
			p-value [adj. p-value] vs. baseline (n)		p-value [adj. p-value] vs. baseline (n)	p-value [adj. p-value] vs. placebo (n)
*Empagliflozin*						
Homoarginine	2.66 ± 3.03	2.45 ± 2.46	0.562 [0.705] (68)	2.11 ± 2.03	0.265 [0.441] (70)	0.085 [0.213] (69)
Arginine	28.39 ± 16.82	26.82 ± 18.37	0.422 [0.613] (68)	26.87 ± 15.59	0.781 [0.863] (69)	0.654 [0.795] (70)
ADMA	52.01 ± 15.30	51.64 ± 14.59	0.280 [0.450] (70)	44.11 ± 14.89	< 0.001 [< 0.001] (71)	< 0.001 [0.001] (70)
SDMA	42.63 ± 12.32	40.80 ± 11.00	0.102 [0.242] (70)	41.07 ± 12.65	0.114 [0.257] (71)	0.759 [0.863] (70)
Creatinine	10.95 ± 3.93	10.57 ± 3.13	0.207 [0.373] (70)	9.62 ± 3.09	< 0.001 [0.001] (71)	0.016 [0.048] (70)
*Dapagliflozin*						
Homoarginine	2.64 ± 5.21	2.58 ± 3.66	0.734 [0.831] (59)	2.07 ± 1.58	0.443 [0.604] (57)	0.984 [0.984] (57)
Arginine	25.67 ± 17.27	30.55 ± 20.44	0.007 [0.053] (59)	32.32 ± 23.70	0.006 [0.053] (58)	0.441 [0.604] (58)
ADMA	47.77 ± 15.51	50.94 ± 16.97	0.174 [0.313] (59)	47.76 ± 17.90	0.923 [0.966] (58)	0.160 [0.313] (58)
SDMA	42.12 ± 12.13	44.85 ± 15.03	0.521 [0.670] (59)	41.87 ± 13.07	0.801 [0.879] (58)	0.172 [0.313] (58)
Creatinine	10.63 ± 3.96	11.15 ± 5.32	0.419 [0.604] (59)	10.22 ± 3.70	0.167 [0.313] (58)	0.069 [0.163] (58)

Data are given in µmol for hArg, Arg, ADMA, and SDMA, for creatinine in mmol, all as mean ± SD; p-values calculated by Wilcoxon signed-rank test, adj. p-values computed based on the procedure of Benjamini and Hochberg¸ p-values refer to the absolute changes in biomarker amount

In terms of amount excreted in urine over 24 h (Ae) hArg is reduced, but not to statistically significant levels. No statistically significant effect is seen for Arg neither.

Ae of ADMA is significantly reduced in the empagliflozin study, but not in the dapagliflozin study. Compared to baseline of the empagliflozin study the mean Ae reduction is 7.90 µmol after empagliflozin (p < 0.001 [adj. p < 0.001]) and after placebo mean Ae was 0.37 µmol lower (p = 0.280 [adj. p = 0.450]). Intraindividual changes in comparison to baseline were − 10.5% for empagliflozin and + 4.1% for placebo (p < 0.001 [adj. p = 0.002]). For dapagliflozin the mean reduction in Ae was 0.01 µmol after dapagliflozin (p = 0.923 [adj. p = 0.966]) and after placebo a mean increase of 3.17 µmol was observed (p = 0.174 [adj. p = 0.313]). Intraindividual changes were + 2.8% after dapagliflozin and + 11.3% after placebo (p = 0.089 [adj. p = 0.190]) as shown in Additional file 1: Fig. S2. Linear mixed effects models likewise attribute a reduction of Ae of ADMA to empagliflozin, but not for dapagliflozin (Additional file 1: Table S5).

Ae of SDMA was not significantly altered in neither of the two trials.

Creatinine excretion was reduced in both trials. For empagliflozin the reduction after empagliflozin from baseline was 1.33 mmol (p < 0.001 [adj. p = 0.001]) and for placebo it was 0.38 mmol (p = 0.207 [adj. p = 0.373]). Intraindividual changes were − 8.1% for empagliflozin and + 3.4% for placebo (p = 0.004 [adj. p = 0.014]). In the dapagliflozin study the mean reduction of Ae for creatinine was 0.41 mmol after dapagliflozin (p = 0.167 [adj. p = 0.313]) compared to baseline. After placebo the mean increase from baseline was 0.52 mmol (p = 0.419 [adj. p = 0.604]). Intraindividual changes were + 0.4% after dapagliflozin and + 9.8% after placebo treatment (p = 0.065 [adj. p = 0.146]). Linear mixed effects models show a significant reduction of Ae of creatinine for empagliflozin, but not for dapagliflozin (Additional file 1: Table S5).

### Renal clearance of biomarkers

A summary of values found for renal clearance for the biomarkers is shown in Table 3 and Additional file 1: Table S6.

**Table 3:** Renal clearance of biomarkers after empagliflozin/dapagliflozin treatment

	At baseline	After placebo		After verum		
			p-value [adj. p-value] vs. baseline (n)		p-value [adj. p-value] vs. baseline (n)	p-value [adj. p-value] vs. placebo (n)
*Empagliflozin*						
Homoarginine	1.147 ± 1.243	1.058 ± 0.920	0.922 [0.965] (68)	1.057 ± 0.955	0.564 [0.705] (70)	0.224 [0.388] (69)
Arginine	0.303 ± 0.232	0.272 ± 0.198	0.148 [0.290] (68)	0.292 ± 0.186	0.998 [0.998] (69)	0.174 [0.326] (70)
ADMA	84.07 ± 26.00	85.74 ± 28.17	0.786 [0.863] (70)	71.01 ± 25.09	< 0.001 [0.001] (71)	0.001 [0.005] (70)
SDMA	81.51 ± 25.14	81.73 ± 24.18	0.956 [0.978] (70)	74.08 ± 25.09	0.004 [0.015] (71)	0.012 [0.039] (70)
Creatinine	116.83 ± 37.52	113.75 ± 33.31	0.129 [0.264] (70)	101.16 ± 31.23	< 0.001 [< 0.001] (71)	0.002 [0.008] (70)
*Dapagliflozin*						
Homoarginine	1.092 ± 1.708	1.167 ± 1.547	0.636 [0.774] (57)	1.077 ± 0.765	0.023 [0.090] (57)	0.431 [0.604] (55)
Arginine	0.258 ± 0.175	0.306 ± 0.222	0.029 [0.093] (57)	0.320 ± 0.206	0.016 [0.090] (58)	0.396 [0.604] (56)
ADMA	75.24 ± 25.75	86.12 ± 33.67	0.009 [0.058] (57)	79.04 ± 32.34	0.498 [0.659] (58)	0.101 [0.216] (56)
SDMA	83.09 ± 31.11	90.68 ± 32.49	0.049 [0.138] (57)	79.89 ± 31.21	0.432 [0.604] (58)	0.019 [0.090] (56)
Creatinine	114.03 ± 41.05	119.36 ± 41.44	0.319 [0.552] (57)	106.38 ± 40.49	0.097 [0.216] (58)	0.024 [0.090] (56)

Data are given in mL/min as mean ± SD, calculated as (c_urine_ * V_urine_) / (c_plasma_ * 1440 min); p-values calculated by Wilcoxon signed-rank test, adj. p-values computed based on the procedure of Benjamini and Hochberg; p-values refer to the absolute changes in biomarker Cl_R_

Similarly to Ae, Cl_R_ of hArg was not significantly altered in the empagliflozin study. In the dapagliflozin study only the linear mixed effects model indicates a statistically significant increase for dapagliflozin (Additional file 1: Table S6).

Cl_R_ of Arg was not significantly altered in the empagliflozin study. In the dapagliflozin study Cl_R_ after both dapagliflozin and placebo treatment was increased, however, the linear mixed effects model indicates no effect of dapagliflozin (Additional file 1: Table S6).

Cl_R_ of ADMA was reduced by 13.06 mL/min after empagliflozin (p < 0.001 [adj. p = 0.001]) and increased by 1.67 mL/min after placebo (p = 0.786 [adj. p = 0.863]) in the empagliflozin trial. The intraindividual percentage changes vs. baseline were − 11.2% for empagliflozin and + 4.2% for placebo (p < 0.001 [adj. p = 0.002]). In the dapagliflozin trial an increase of 3.80 mL/min after dapagliflozin (p = 0.498 [adj. p = 0.659]) compared to baseline and after placebo an increase of 10.88 mL/min (p = 0.009 [adj. p = 0.058]) was found. The intraindividual change was + 9.1% after dapagliflozin and + 24.1% after placebo with a p-value of 0.054 [adj. p = 0.144] between them. The linear mixed effects models indicate a statistically significant effect for empagliflozin, but not for dapagliflozin (Additional file 1: Table S6).

In the empagliflozin trial Cl_R_ of SDMA was reduced by 7.43 mL/min after empagliflozin (p = 0.004 [adj. p = 0.015]) and increased by 0.22 mL/min after placebo (p = 0.956 [p = 0.978]). The corresponding mean intraindividual changes were − 4.6% for empagliflozin and + 6.3% for placebo (p = 0.008 [adj. p = 0.024]). In the dapagliflozin trial Cl_R_ was reduced by 3.20 mL/min after dapagliflozin (p = 0.432 [adj. p = 0.604]) and increased by 7.59 mL/min after placebo (p = 0.049 [adj. p = 0.138]). Intraindividually the change from baseline was + 0.5% for dapagliflozin and + 17.5% for placebo (p = 0.012 [adj. p = 0.066]). The linear mixed effects models indicate a statistically significant reduction for both drugs (Additional file 1: Table S6).

Cl_R_ of creatinine in the empagliflozin trial was reduced by 15.67 mL/min after empagliflozin (p < 0.001 [adj. p < 0.001]) and 3.08 mL/min after placebo (p = 0.129 [adj. p = 0.264]). Mean intraindividual changes were -9.6% for empagliflozin and + 3.0% for placebo (p = 0.001 [adj. p = 0.005]). The mean change from baseline in the dapagliflozin study was a reduction of 7.65 mL/min for dapagliflozin (p = 0.097 [adj. p = 0.216]) and an increase of 5.33 mL/min for placebo (p = 0.319 [adj. p = 0.552]). The intraindividual changes were − 1.8% for dapagliflozin and + 12.2% for placebo (p = 0.025 [adj. p = 0.090]). The linear mixed effects models indicate a statistically significant reduction of creatinine Cl_R_ for both drugs compared to placebo (Additional file 1: Table S6).

### Intraindividual correlation of the treatment related change for empagliflozin

A weak inverse correlation between SDMA and SBP (r = − 0.279, p = 0.019) within patient effects was found for empagliflozin treatment in comparison to baseline. The other biomarkers did not correlate significantly with intraindividual changes in HbA1c, FPG, BMI, or SBP. Dapagliflozin treatment showed correlations of within patient effects of hArg and Arg with SBP (r = 0.278, p = 0.033 and r = 0.366, p = 0.004, respectively) and SDMA with FPG (r = − 0.259, p = 0.048). After exclusion of two outliers in the intraindividual changes of SBP, the correlation between hArg and Arg is no longer statistically significant (r = 0.234, p = 0.080 and r = 0.260, p = 0.051, respectively). See Table 4 and Additional file 1: Table S2 for more details.

**Table 4:** Intraindividual correlations of the treatment related change after empagliflozin or dapagliflozin treatment in comparison to baseline

	Glycated hemoglobin		Fasting plasma glucose		Body mass index		Systolic blood pressure	
	r	p-value	r	p-value	r	p-value	r	p-value
*Empagliflozin*								
Homoarginine	0.041	0.736	0.005	0.969	− 0.079	0.513	0.037	0.758
Arginine	− 0.204	0.088	− 0.116	0.336	− 0.081	0.502	0.044	0.715
ADMA	− 0.171	0.153	− 0.010	0.931	− 0.142	0.236	− 0.134	0.266
SDMA	− 0.175	0.144	− 0.042	0.725	− 0.007	0.952	− 0.279	0.019
*Dapagliflozin*								
Homoarginine	− 0.104	0.432	− 0.210	0.110	0.020	0.882	0.278	0.033
Arginine	− 0.186	0.159	− 0.195	0.139	− 0.066	0.619	0.366	0.004
ADMA	− 0.110	0.407	− 0.182	0.168	− 0.127	0.337	0.073	0.581
SDMA	− 0.075	0.571	− 0.259	0.048	0.012	0.928	− 0.073	0.583

Correlations are shown as Pearson correlation coefficients (r)

## Discussion

In the present study we evaluated the effects of treatment with empagliflozin and dapagliflozin on Arg-related risk markers in order to identify effects of SGLT-2 inhibitors in T2D that extend beyond glucose control. In plasma hArg was significantly reduced and SDMA increased after treatment with both SGLT-2 inhibitors. The Cl_R_ for SDMA, ADMA, and creatinine was significantly reduced (for ADMA in the empagliflozin trial only), whereas Cl_R_ for hArg and Arg was not significantly altered.

The main findings in changes of the biomarkers, namely increase of SDMA and reduction of hArg in plasma, show a high degree of similarity in responses to treatment for both empagliflozin and dapagliflozin. This suggests, that the observed effects are not specific to either drug, but rather can be considered as group effects for SGLT-2 inhibitors. At first sight, the observed changes in the plasma concentrations of hArg and SDMA may not appear clinically relevant. It has to be kept in mind, however, that rather subtle differences in plasma concentrations of these biomarkers are related to substantial differences in long-term overall mortality [12, 18–20]. Considering known beneficial effects of the SGLT-2 inihibitors on cardiovascular outcomes on one hand and the adverse outcomes associated with low plasma hArg and elevated plasma SDMA on the other hand, the observed effects of SGLT-2 inhibitors on these biomarkers were somewhat unexpected. Irrespective of any other considerations these data indicate that in patients taking empagliflozin or dapagliflozin risk estmates based on hArg or SDMA should be regarded with caution until the prognostic relevance of these risk markers has been reassessed for this patient group.

Reports of marked effects of SGLT-2 inhibitors on survival, cardiovascular events and renal disease have sparked a search for underlying mechanisms beyond improved glycaemic control and compensatory metabolic changes related to SGLT-2 induced energy and water loss [26]. The diuretic effects of SGLT-2 inhibition may affect the renal handling of Arg-related biomarkers, as it is demonstrated by a reduced Cl_R_ of creatinine we observed in accordance with other studies [27, 28]. Arg is a key substrate for various pathways including vascular Arg-NO-signalling and it also affects insulin signalling in diabetes [29–31]. The structurally related hArg is regarded as a protective factor for the cardio- and cerebrovascular system and the kidneys [10–14]. In contrast ADMA and SDMA are considered risk markers with possible causal components such as inhibition of nitric oxide synthase for ADMA, and competition for several mechanisms shared with Arg and hArg [10, 16, 17, 32]. ADMA and SDMA are primarily derived from protein catabolism, their elimination is dominated by metabolism (ADMA) and renal excretion (SDMA), respectively [33–36]. Due to their chemical relatedness hArg, Arg, ADMA, and SDMA share key transport mechanisms in the kidney and several metabolic pathways, while they distinctly differ in others [32, 37]. These differences in metabolic and renal handling of the Arg-related biomarkers (more details are provided in Additional file 1: Table S3) likely contribute to the observed effects of SGLT-2 inhibitors on these markers.

The plasma concentrations of hArg, Arg, ADMA, and SDMA found in T2D patients in both studies at baseline are within the range also found in healthy subjects, however, they are at the lower end of the reference ranges as summarized by Banjarnahor et al. [32](Table 1).

A recent untargeted analysis of effects on the metabolic signature of empagliflozin treatment in plasma of 25 T2D patients found signals for some of the biomarkers we studied [38]. In that study, both hArg and Arg were reduced, but only Arg was qualified as significantly altered. In contrast, in our studies the numerical decrease of Arg plasma concentration is not significant but we found a highly significant reduction of hArg concentrations. The methodology used by Kappel et al*.* most likely could technically not differentiate between ADMA and SDMA, and they did not observe a significant effect for the combined signal of ADMA/SDMA. However, plasma concentrations were slightly elevated for the combined signal of ADMA and SDMA. The method we used in the present study is able to differentiate between the signals for ADMA and SDMA and showed diverging SGLT-2 inhibitor effects in plasma. In our analysis ADMA was not significantly changed in plasma, but the change for SDMA was highly significant. Of note, Cl_R_ of SDMA was reduced with increased plasma concentrations of SDMA not paralleled by an increased Ae in urine. As renal excretion is the main route of elimination for SDMA, the reduced Cl_R_ serves as an explanation for its increase in plasma concentration [33, 34].

The plasma concentration of ADMA was not altered by SGLT-2 inhibition, while Cl_R_ of ADMA was significantly reduced in the empagliflozin study. In the dapagliflozin group a Cl_R_ reduction of ADMA by verum treatment in comparison to placebo is indicated by the linear mixed effects model, however, this is not congruent with the other analysis performed in the present study. Augmented alanine-glyoxylate aminotransferase 2 metabolism could explain steady ADMA and even lowered hArg, but is not compatible with increased SDMA concentrations. This leaves augmented metabolism of ADMA via dimethylarginine dimethylaminohydrolase 1 compensating for reduced renal excretion of ADMA [35, 36], as a plausible explanation and possible beneficial effect of SGLT-2 inhibition with empagliflozin, but it is unclear if the same is applicable to dapagliflozin. Technical explanations, such as differences in samples size (n = 70 for empagliflozin vs. n = 56 dapagliflozin), aside, it cannot be ruled out that this observation is due to differential impact of the drugs on renal clearance of ADMA.

We found no relevant changes for plasma concentration, Cl_R_, or Ae for Arg, which could be attributed to masking of any drug effects by exogenous factors such as variable intake with diet. Nutrition was not controlled throughout both trials investigated here. Of the biomarkers assessed, Arg is likely the most sensitive to dietary effects [39]. Therefore, large intraindividual variations may cause statistical calculations not to be significant. In contrast to Arg, the homologue hArg stems predominantly from endogenous biosynthesis [40]. Considering its presumed protective role in CVD and/or CKD a decrease in plasma concentration of hArg was unexpected [11–14]. However, its plasma concentration is also known to be inversely related to glomerular filtration [11–13]. A further possible explanation is a modified energy metabolism in the kidney. Reduced re-absorption of glucose fundamentally alters energy metabolism in the kidney, leading to, amongst others, increased use of lysine for biosynthesis of ketone bodies [26, 38]. Renal biosynthesis via the enzyme arginine:glycine amidinotransferase requires lysine as a substrate and is regarded as the main source of hArg [37, 41]. Hence, lack of lysine may explain a decrease in hArg plasma concentrations. This hypothesis would need further investigations, among others quantification of lysine, which was beyond the scope of this study. Taken together, the non-significant reduction of Ae of hArg in urine can be regarded as a consequence of the reduced concentration in plasma, which is reflected by the largely unaffected Cl_R_ of hArg.

Finally, changes in plasma concentrations of the biomarkers after empagliflozin or dapagliflozin treatment do not constantly correlate with intraindividual changes of clinical parameters affected by SGLT-2 inhibitors (Table 4), implying that the effects are rather due to SGLT-2 inhibition, than just secondary effects due to changes of glycaemic control and disease status. The inverse correlation between SDMA and SBP in the empagliflozin group and FPG in the dapagliflozin group is likely to be coincidental with the effect of SGLT-2 inhibition on both. Correlations between the intraindividual changes of both hArg and Arg and SBP in the dapagliflozin group may be caused by two outliers. For two participants of the dapagliflozin study the SBP increased by 27 and 20 mm Hg, respectively, after verum treatment compared to baseline. Re-analysis without these outliers indicates that the correlations of intraindividual SBP change with hArg and Arg are no longer statistically significant. We did not further investigate this observation as this was not the main focus of the present study. Future studies may investigate if these correlations are coincidental or if non-reduction of SBP correlates with changes in plasma concentrations of hArg or Arg in a similar way.

## Conclusions

The SGLT-2 inhibitors empagliflozin and dapagliflozin affect plasma concentrations and/or renal handling of Arg-related risk-markers, which may also affect risk estimates based on these factors. The pattern of the observed effects on the different biomarkers was unexpected and does not provide a direct explanation (i.e. causal links) for beneficial effects of the SGLT-2 inhibitors on CVD and CKD, as the protective marker hArg was reduced and the deleterious marker SDMA was increased in plasma. However, the observed effects may guide future investigations such as an exploration of a potential beneficial impact of hArg supplementation for T2D patients under therapy with an SGLT-2 inhibitor.

## Supplementary Information

**Additional file 1.** Additional figures and tables.

## Data Availability

The datasets used and analysed during the current study are available from the corresponding author on reasonable request.

## Abbreviations

ADMA: Asymmetric dimethylarginine
Ae: Amount excreted in urine over 24 h
Arg: l-arginine
BMI: Body mass index
CKD: Chronic kidney disease
Cl_R_: Renal clearance
CVD: Cardiovascular disease
FPG: Fasting plasma glucose
hArg: l-homoarginine
HbA1c: Glycated hemoglobin
HCl: Hydrochloride salt
LC/MS: Liquid chromatography–mass spectrometry
LC–MS/MS: Liquid chromatography–mass spectrometry/mass spectrometry
MRM: Multiple reaction monitoring
MS: Mass spectrometry
SBP: Systolic blood pressure
SDMA: Symmetric dimethylarginine
SGLT-2: Sodium-glucose cotransporter 2
T2D: Type 2 diabetes mellitus
