# Supplementary material for: Effects of treatment with SGLT-2 inhibitors on arginine-related cardiovascular and renal biomarkers
Source: Cardiovasc Diabetol. 2022 Jan 6;21:4. doi: 10.1186/s12933-021-01436-x (PMC8740418; doi:10.1186/s12933-021-01436-x)
Supplement: Supplementary file 1 — Additional file 1. Additional figures and tables. [file 12933_2021_1436_MOESM1_ESM.pdf]

SUPPLEMENTAL INFORMATION

Effects of Treatment with SGLT-2 Inhibitors on Arginine-related Cardiovascular and Renal Biomarkers

Arne Gessner[1], Anna Gemeinhardt[1], Agnes Bosch[2], Dennis Kannenkeril[2], Christian Staerk[3], Andreas Mayr[3], Martin F. Fromm[1], Roland E. Schmieder[2], and Renke Maas[1]

[1]Institute of Experimental and Clinical Pharmacology and Toxicology, Friedrich-Alexander-Universität Erlangen-Nürnberg, Erlangen, Germany

[2]Department of Nephrology and Hypertension, University Hospital Erlangen, Germany

[3]Institute of Medical Biometry, Informatics and Epidemiology, University Hospital Bonn, Bonn, Germany

Address for correspondence: Dr. Arne Gessner, Institute of Experimental and Clinical Pharmacology and Toxicology, Friedrich-Alexander-Universität Erlangen-Nürnberg, Fahrstr. 17, 91054 Erlangen, Germany. Tel: +49 9131 85 22869; Fax: +49 9131 85 22773; E-mail: arne.gessner@fau.de

Table S1: Results for quality control (QC) samples

A total of 386 plasma and 385 urine samples were quantified in 12 batches (6 plasma, 6 urine). Each batch contained QC samples at the nominal concentration indicated in duplicates. An aliquot of a real sample (LTS) was analyzed in each batch to ensure consistency.

**Homoarginine**						
	Plasma			Urine		
	NC	Acc.	Prec.	NC	Acc.	Prec.
Q1	20	104.4	7.0	10	95.7	10.2
Q5	4	98.9	7.6	1.48	95.8	8.3
Q9	0.8	100.1	6.8	0.0412	107.6	9.5
Q10	0.4	101.8	8.0	n.a. (below validated range)		
LTS female	n.a.		8.8	n.a.		13.4
LTS male	n.a.		8.6	n.a.		10.9
**Arginine**						
	Plasma			Urine		
	NC	Acc.	Prec.	NC	Acc.	Prec.
Q1	300	103.2	5.1	100	105.7	12.2
Q5	60	99.8	9.9	28.4	104.4	9.5
Q9	12	94.9	7.5	2.5	96.1	14.1
Q10	6	97.8	11.4	1.67	99.6	14.0
LTS female	n.a.		10.2	n.a.		11.6
LTS male	n.a.		12.2	n.a.		7.8
**ADMA**						
	Plasma			Urine		
	NC	Acc.	Prec.	NC	Acc.	Prec.
Q1	5	106.3	4.2	100	101.7	9.2
Q5	1	99.9	4.0	28.4	98.2	10.8
Q9	0.2	101.3	7.0	2.5	101.5	7.7
Q10	0.1	104.6	6.6	1.67	100.4	12.5
LTS female	n.a.		4.0	n.a.		9.3
LTS male	n.a.		3.6	n.a.		7.3
**SDMA**						
	Plasma			Urine		
	NC	Acc.	Prec.	NC	Acc.	Prec.
Q1	5	105.3	4.5	100	100.9	5.3
Q5	1	100.7	3.2	28.4	102.2	6.7
Q9	0.2	96.5	5.5	2.5	100.7	7.7
Q10	0.1	97.5	5.2	1.67	96.3	6.9
LTS female	n.a.		5.2	n.a.		6.7
LTS male	n.a.		2.7	n.a.		3.8
**Creatinine**						
	Plasma			Urine		
	NC	Acc.	Prec.	NC	Acc.	Prec.
Q1	350	105.1	4.7	50000	105.9	7.0
Q5	70	94.1	4.7	14200	104.3	5.4
Q9	14	105.4	10.2	1250	96.5	11.8
Q10	n.a.			417	96.6	6.9
LTS female	n.a.		3.4	n.a.		7.1
LTS male	n.a.		3.1	n.a.		6.8

NC: nominal concentration in µmol/L, Acc.: accuracy in % of NC, Prec.: precision as coefficient of variation of acc. in %, LTS: long term stability.

Table S2: Clinical characteristics after 6 weeks of treatment with empagliflozin or dapagliflozin

	Empagliflozin			Dapagliflozin		
	At baseline	After verum	p-value	At baseline	After verum	p-value
**HbA1c**	6.75 ± 0.78	6.70 ± 0.75	0.335	6.67 ± 0.73	6.62 ± 0.67	0.224
**FPG**	139.6 ± 31.1	116.8 ± 19.6	< 0.001	132.3 ± 28.1	114.0 ± 19.1	< 0.001
**BMI**	30.1 ± 4.2	29.8 ± 4.2	< 0.001	29.9 ± 4.3	29.5 ± 4.1	< 0.001
**SBP**	128.8 ± 13.9	123.1 ± 12.5	< 0.001	133.3 ± 12.5	129.4 ± 11.8	0.009

HbA1c: glycated hemoglobin in %, FPG: fasting plasma glucose in mg/dL, BMI: body mass index in kg/m^2, SBP: systolic blood pressure in mmHg; all in mean ± standard deviation.

Table S3: Key mechanisms governing transport and metabolism of homoarginine, arginine, ADMA, and SDMA (adopted from [1])

	Homoarginine	Arginine	ADMA	SDMA
**CAT1**	Transported	Transported	Transported	Transported
**DDAH1**	No substrate	No substrate	Main metabolizing enzyme	No substrate
**AGXT2**	Substrate		Substrate	Main metabolizing enzyme
**Arginases**	No substrate	Substrate	No substrate	No substrate
**Nitric oxide synthases**	Weak substrate	Major Substrate	Inhibitor	No substrate
**Urine**	Glomerular filtration but extensive tubular reabsorbtion	Glomerular filtration but extensive tubular reabsorbtion	Glomerular filtration, enriched in urine, secondary route of elimination	Glomerular filtration, enriched in urine, major route of elimination

Table S4: Effect estimates of the difference in plasma concentrations between verum and placebo treatment. Linear mixed effects models for the two post-baseline concentrations (verum / placebo), adjusted for baseline, the ordering in the cross-over design and the additional covariates age and sex. The models contain subject-specific random intercepts to account for repeated measurements.

		Difference after verum treatment vs. placebo treatment	95% confidence interval of difference		p-value
			lower limit	upper limit	
Empagliflozin	Homoarginine	-0.255	-0.375	-0.134	< **0.001**
	Arginine	-3.41	-7.64	0.81	0.118
	ADMA	0.007	-0.009	0.023	0.365
	SDMA	0.040	0.028	0.053	< **0.001**
	Creatinine	1.56	0.06	3.07	**0.046**
Dapagliflozin	Homoarginine	-0.211	-0.322	-0.099	**0.001**
	Arginine	0.10	-3.74	4.00	0.959
	ADMA	0.008	-0.009	0.024	0.365
	SDMA	0.026	0.013	0.040	< **0.001**
	Creatinine	2.42	0.026	4.84	0.054

Table S5: Effect estimates of the difference in urine concentration between verum and placebo treatment. Linear mixed effects models for the two post-baseline concentrations (verum / placebo), adjusted for baseline, the ordering in the cross-over design and the additional covariates age and sex. The models contain subject-specific random intercepts to account for repeated measurements. Outcome values were log-transformed.

		Difference after verum treatment vs. placebo treatment	95% confidence interval of difference		p-value
			lower limit	upper limit	
Empagliflozin	log Homoarginine	-0.06	-0.24	0.11	0.479
	log Arginine	0.03	-0.13	0.19	0.703
	log ADMA	-0.21	-0.33	-0.08	**0.002**
	log SDMA	-0.01	-0.07	0.06	0.841
	log Creatinine	-0.11	-0.18	-0.03	**0.007**
Dapagliflozin	log Homoarginine	0.07	-0.10	0.26	0.423
	log Arginine	0.01	-0.18	0.20	0.897
	log ADMA	-0.07	-0.16	0.01	0.093
	log SDMA	-0.06	-0.15	0.03	0.179
	log Creatinine	-0.08	-0.16	0.004	0.066

Table S6: Effect estimates of the difference in renal clearance between verum and placebo treatment. Linear mixed effects models for the two post-baseline concentrations (verum / placebo), adjusted for baseline, the ordering in the cross-over design and the additional covariates age and sex. The models contain subject-specific random intercepts to account for repeated measurements. Outcome values were log-transformed.

		**Difference after verum treatment vs. placebo treatment**	**95% confidence interval of difference**		**p-value**
			**lower limit**	**upper limit**	
Empagliflozin	log Homoarginine	0.104	-0.082	0.290	0.276
	log Arginine	0.108	-0.065	0.282	0.228
	log ADMA	-0.232	-0.383	-0.082	**0.004**
	log SDMA	-0.113	-0.190	-0.036	**0.006**
	log Creatinine	-0.125	-0.198	-0.052	**0.001**
Dapagliflozin	log Homoarginine	0.194	0.016	0.375	**0.038**
	log Arginine	0.016	-0.179	0.209	0.869
	log ADMA	-0.097	-0.201	0.007	0.073
	log SDMA	-0.140	-0.240	-0.041	**0.008**
	log Creatinine	-0.122	-0.217	-0.029	**0.014**

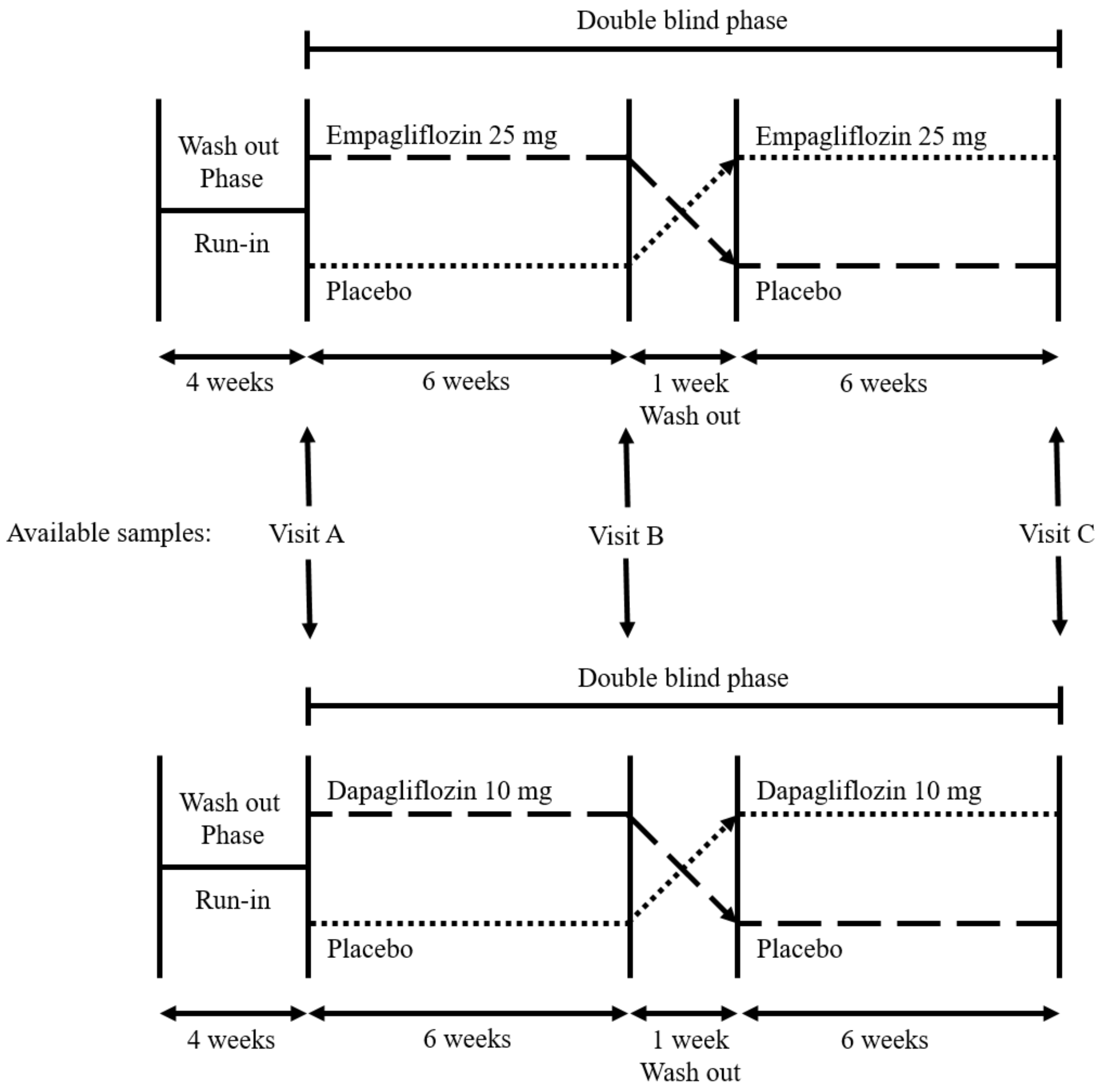

Figure S1: Study design. Plasma and urine samples were taken at the indicated time points (adopted from [2]).

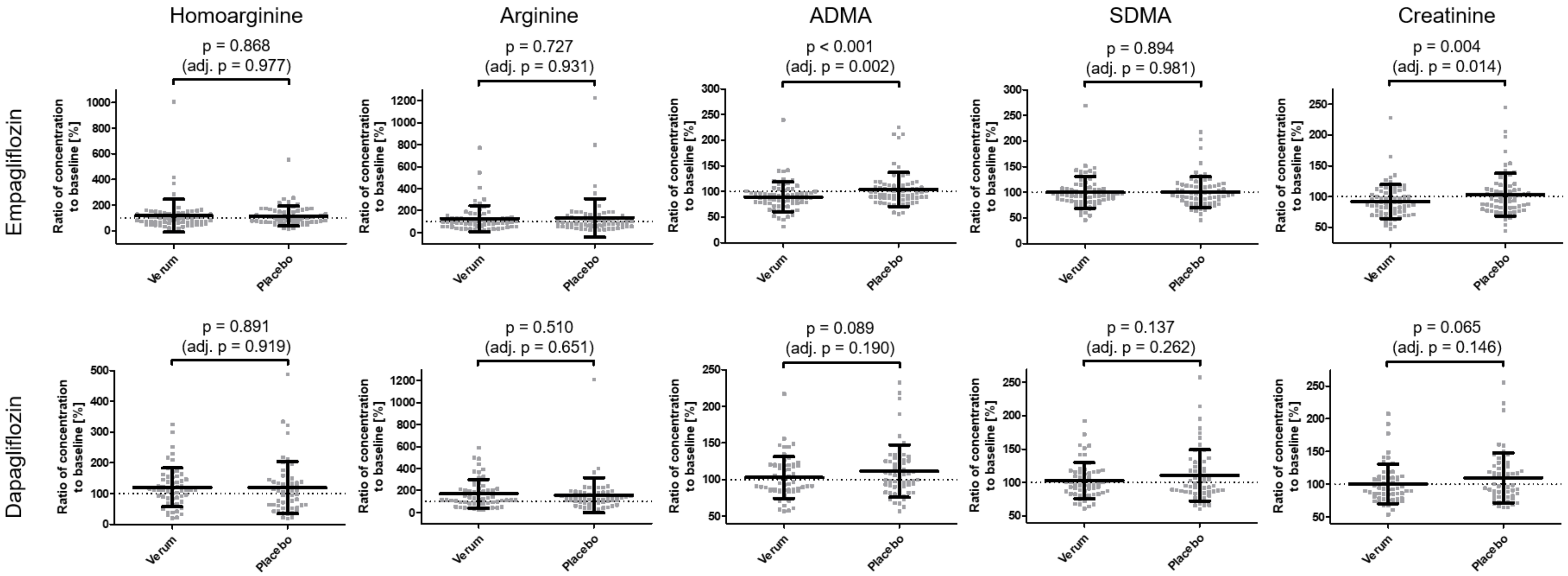

Figure S2: Intraindividual percental change of amount excreted in urine after treatment with empagliflozin or dapagliflozin, p-values calculated by Wilcoxon signed-rank test; p-values refer to the percentage changes of biomarkers; adj. p-values computed based on the procedure of Benjamini and Hochberg.

SUPPLEMENTARY REFERENCES

1. Banjarnahor S, Rodionov RN, König J, Maas R: Transport of L-arginine related cardiovascular risk markers. *J Clin Med.* 2020; 9(12):3975-4016.
2. Ott C, Jumar A, Striepe K, Friedrich S, Karg MV, Bramlage P, Schmieder RE: A randomised study of the impact of the SGLT2 inhibitor dapagliflozin on microvascular and macrovascular circulation. *Cardiovasc Diabetol.* 2017; 16(1):26-35.
